# Supplementary material for: Hyperpolarized 15N-labeled, deuterated tris(2-pyridylmethyl)amine as an MRI sensor of freely available Zn2+
Source: Commun Chem. 2020 Dec 9;3:185. doi: 10.1038/s42004-020-00426-6 (PMC8244538; doi:10.1038/s42004-020-00426-6)
Supplement: Supplementary file 1 — Supplementary Information [file 42004_2020_426_MOESM1_ESM.pdf]

Hyperpolarized ^{15}N-labeled, deuterated tris(2-pyridylmethyl)amine as an MRI sensor of freely available Zn^{2+}

Eul Hyun Suh[1], Jae Mo Park[1,2,3], Lloyd Lumata[4], A. Dean Sherry[1,2,5], Zoltan Kovacs[1,5*]

[1] Advanced Imaging Research Center, University of Texas Southwestern Medical Center, Dallas, TX, 75390, USA

[2] Department of Radiology, University of Texas Southwestern Medical Center, Dallas, TX, USA

[3] Department of Electrical and Computer Engineering, University of Texas at Dallas, Richardson, TX, 75080, USA

[4] Department of Physics, University of Texas Dallas, Richardson, TX, 75080, USA

[5] Department of Chemistry and Biochemistry, University of Texas Dallas, Richardson, TX, 75080, USA

*** Correspondence:** zoltan.kovacs@utsouthwestern.edu

Contents

1. Supplementary Methods. Quantification of Zn^{2+} concentration S2
2. Supplementary Equation 1. Calculation of Zn^{2+} concentration from the HP-^{15}N NMR data S2
3. Supplementary Equation 2. The thermodynamic stability constant K_{ZnTPA} S2
4. Supplementary Table 1. The T_1 value of [^{15}N]TPA and [^{15}N]TPA-d_6 .... S2
5. Supplementary Figure 1. ^{15}N DNP NMR experiments with unlabeled TPA S3
6. Supplementary Figure 2. ^{15}N DNP-NMR experiments with [^{15}N]TPA S3
7. Supplementary Figure 3. Zn^{2+} binding selectivity and stoichiometry of TPA S4
8. Supplementary Figure 4. Time-dependent ^{15}N NMR spectra of BPH tissue S4

Supplementary Methods.

Quantification of Zn^{2+}concentration.

Determination of the Zn-concentration from the peak areas of the of the ^{15}N signal of free TAP and the Zn-TPA complex is based on the assumption that TPA reacts quantitatively with Zn^{2+}. Thus, knowing the TPA concentration, the Zn-concentration can be calculated the as follows:

$$[Zn^{2+}] = \frac{A_{Zn^{2+}-TPA-d_6}}{A_{TPA-d_6} + A_{Zn^{2+}-TPA-d_6}} \times [TPA - d_6] \qquad \text{Supplementary Eq. 1}$$

A_{TPA-d_6} : Peak area of the ^{15}N signal of [^{15}N]TPA-d_6 (40 ppm) in the first spectrum
$A_{Zn^{2+}-TPA-d_6}$: Peak area of the ^{15}N signal of Zn^{2+}-[^{15}N]TPA-d_6 complex (20 ppm) in the first spectrum
$[TPA - d_6]$: Total concentration of HP- $TPA - d_6$

Supplementary Eq.1 can be used to calculate the Zn-concentration in the concentration range down to about 1 nM (ignoring the detecting limit of ^{15}N NMR). Below this value the dissociation of the ZnTPA complex cannot be neglected (K_{ZnTPA} = 11). The thermodynamic stability constant K_{ZnTPA} is expressed as Supplementary Equation 2:

$$TPA \quad + \quad Zn^{2+} \rightleftharpoons ZnTPA$$

$$K_{ZnTPA} = \frac{[ZnTPA]}{[TPA][Zn^{2+}]} \qquad \text{Supplementary Eq. 2}$$

The dissociation constant is defined as the inverse of the stability constant: $K_{D\ ZnTPA} = K_{ZnTPA}^{-1}$. In the concentration range, where the K_{DZnTPA} approximately matches the level of Zn^{2+}, the Zn^{2+} concentration can quantitatively be determined by measuring the ratio of [TPA-Zn]/[TPA] independently of the total concentration of HP-TPA.

Supplementary Table 1. The T_1 value of [^{15}N]TPA and [^{15}N]TPA-d_6 under the different conditions

Compound	T_1 (s)	Field (T)
^{15}N-TPA	25	$9.4^{a,c}$
^{15}N-TPA-d_6	70	$9.4^{a,c}$
^{15}N-TPA-d_6	89	$3^{b,d}$
^{15}N-TPA-d_6	79	$1^{a,c}$

[a] Hypersense; [b] SPINlab; [c] in the presence of ProHance (2 mM); [d] in the absence of ProHance

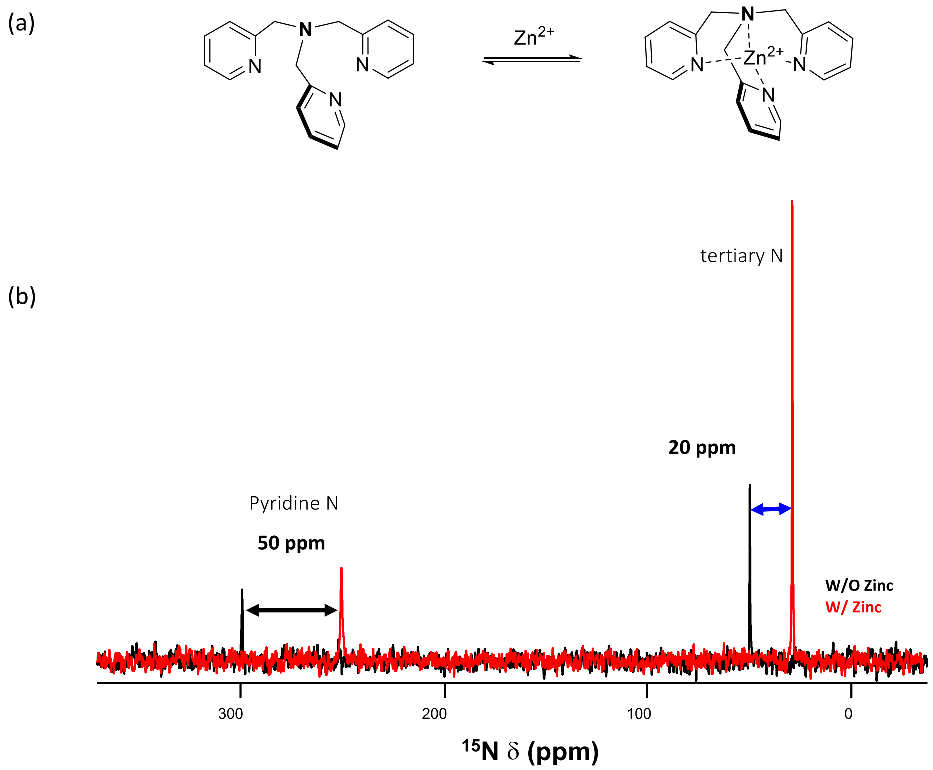

Supplementary Figure 1. ^{15}N DNP NMR experiments with unlabeled TPA. (a) Scheme for Zn^{2+} sensing with TPA. (b) The first ^{15}N spectrum of hyperpolarized unlabeled TPA in the absence (spectrum in black) and presence of 40 mM Zn^{2+} (spectrum in red), (pH = 6.8).

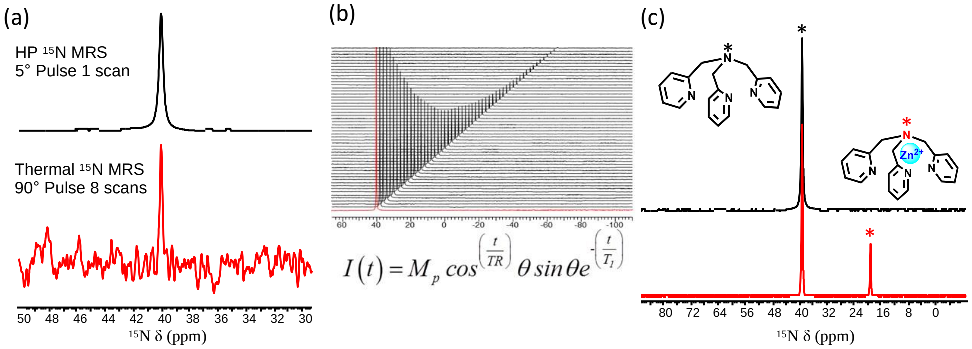

Supplementary Figure 2. ^{15}N DNP-NMR experiments with [^{15}N]TPA. (a) ^{15}N NMR spectrum of hyperpolarized and thermal equilibrium [^{15}N]TPA at 9.4 T, 298 K. (b) Decay of the HP-^{15}N magnetization of HP-[^{15}N]TPA. (c) ^{15}N NMR chemical shift of ^{15}N-TPA in the absence and presence of Zn^{2+} (0.25 eq) (pH = 6.8).

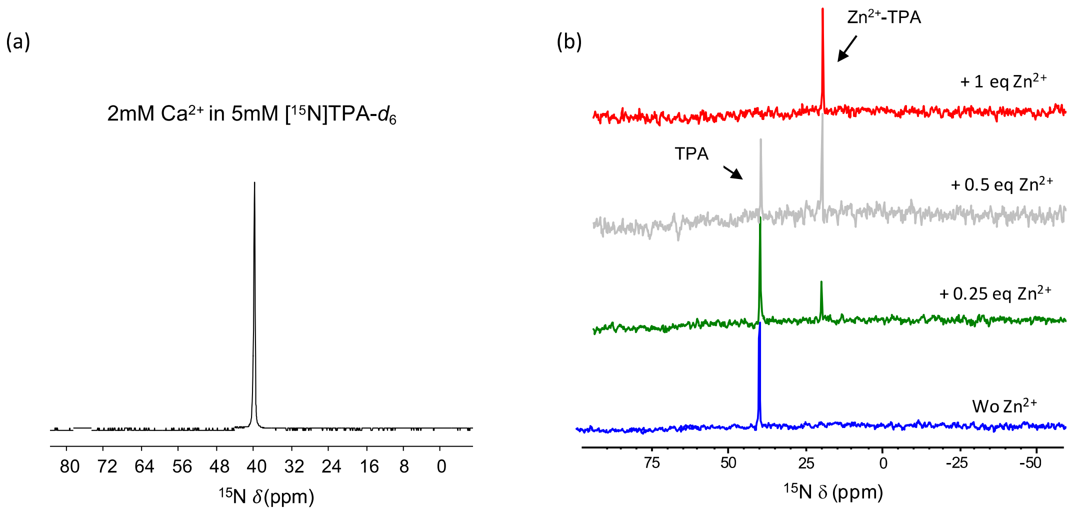

Supplementary Figure 3. Zn^{2+}-binding selectivity and stoichiometry of [^{15}N]TPA. (a) Single-scan ^{15}N spectrum in the presence of Ca^{2+}. (b) Thermal ^{15}N NMR spectroscopy in the presence of various Zn^{2+} concentrations (pH = 6.8).

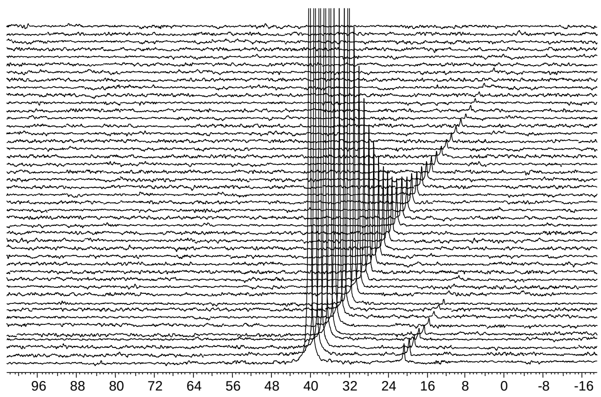

Supplementary Figure 4. Time-dependent ^{15}N NMR spectra of BPH tissue collected by adding 4.2 mM HP-[^{15}N]TPA-d_6 (9.4 T, 298 K and pH 7.0).
